# Anaplastic Lymphoma Kinase Rearrangement-Positive Pulmonary Adenocarcinoma With Scattered Bilateral Breast Metastases: A Case Report

**DOI:** 10.7759/cureus.80325

**Published:** 2025-03-10

**Authors:** Shinichi Matsuzaki, Ichiro Inoshima, Yasutaka Hirasawa, Yoshio Horii, Sumihito Nobusawa

**Affiliations:** 1 Respiratory Medicine, Tatebayashi Kosei Hospital, Tatebayashi, JPN; 2 Pulmonary Medicine, Shinmatsudo Central General Hospital, Shinmatsudo, JPN; 3 Breast Surgery, Tatebayashi Kosei Hospital, Tatebayashi, JPN; 4 Human Pathology, Gunma University Graduate School of Medicine, Maebashi, JPN

**Keywords:** anaplastic lymphoma kinase (alk), breast metastases, fluorescence in situ hybridization (fish), immunohistochemistry, lung cancer

## Abstract

A 67-year-old woman was referred to our hospital because of chest X-ray abnormalities. Chest computed tomography revealed a lobulated lung mass in the right lower lobe and multiple scattered nodules in both breasts. The lung mass was diagnosed as anaplastic lymphoma kinase (ALK) rearrangement-positive pulmonary adenocarcinoma through a transbronchial biopsy. Histological findings of bilateral needle biopsy of breast nodules were nearly identical to that of lung nodule, and fluorescence in situ hybridization showed ALK rearrangement. These results confirmed the diagnosis of ALK rearrangement-positive pulmonary adenocarcinoma with bilateral scattered breast metastases and its metastatic form of lung adenocarcinoma, which is rare.

## Introduction

Lung cancer predominantly metastasizes to the brain, liver, and bones, whereas metastases to the breast are infrequent. Bilateral breast metastases are even rarer. Indeed, the echinoderm microtubule-associated protein-like 4 (EML4)-anaplastic lymphoma kinase (ALK) fusion gene is expressed in patients with a low incidence of pulmonary adenocarcinoma, reported in 4%-6% of patients with non-small cell lung cancer (NSCLC) [1].

On the contrary, among malignant breast tumors, the incidence of metastatic breast tumors is low, ranging from 0.2% to 1.4% [2-4], and multiple breast metastasis is even rarer. The most common primary sources of breast metastases are malignant melanoma, lung cancer, and gynecological cancers [5]. Hence, differentiating between lung cancer mammary metastases and primary breast cancer is potentially crucial.

Herein, we report a rare case of bilateral scattered breast metastases of lung adenocarcinoma with ALK rearrangement, which required differentiation from invasive breast cancer by immunohistochemistry (IHC) and fluorescence in situ hybridization (FISH).

## Case presentation

**Figure 1:**
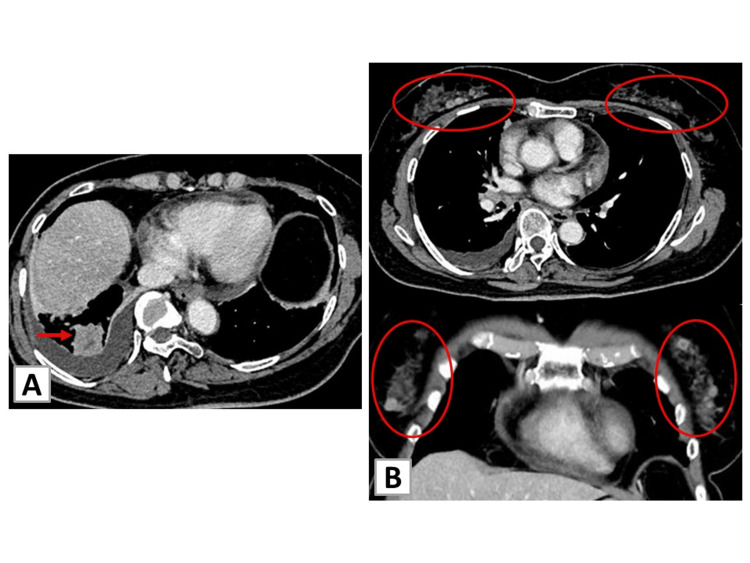
Chest CT findings at initial presentation (A) CT showing a nodule in the lower right lobe (arrow) and accumulation of pleural effusion. (B) CT in axial (upper) and coronal (bottom) views showing scattered nodules in bilateral breasts (red circles). CT, computed tomography

A 67-year-old woman was referred to our hospital due to a suspicion of right pleural effusion on chest X-ray imaging. She had a persistent cough for approximately one month. Her medical history included bipolar disorder, sleep apnea syndrome, and hypertension, which were well-controlled with adequate medicine. She was never a smoker. Vital signs and physical features on presentation were normal. The carbohydrate antigen 19-9 (CA19-9) level was 282 U/mL. Computed tomography (CT) revealed a 3.1 cm right lower lobe tumor, lymphadenopathy, right pleural effusion, and pericardial effusion (Figure 1).

Moreover, approximately 20 scattered tumor shadows, measuring up to 1.2 cm, were also observed in both breasts.

**Figure 2:**
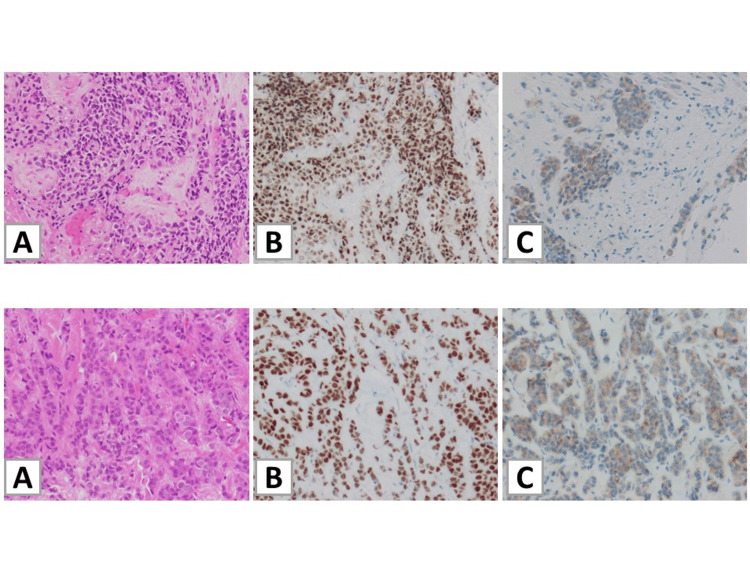
Histological findings from the biopsy specimens of the lung and breast Biopsy specimens of the lung (upper panel) and breast (bottom panel). (A) Findings of poorly differentiated adenocarcinoma on hematoxylin and eosin staining. (B) Immunohistopathological analysis demonstrating positive staining for TTF-1. (C) Immunohistopathological analysis demonstrating positive staining for ALK. Original magnification x200. ALK, anaplastic lymphoma kinase; TTF-1, thyroid transcription factor-1

Molecular analysis of the transbronchial lung biopsy of lung adenocarcinoma specimen was performed using a board next-generation sequencing panel, Oncomine Dx Target Test (ODxTT, Thermo Fisher Scientific, Waltham, MA) Multi-CDx System, and EML4-ALK fusion gene was detected. Hematoxylin and eosin (HE) staining of the specimen revealed positivity for AE1/AE3, CAM5.2, E-cadherin, and thyroid transcription factor-1 (TTF-1) according to the IHC analysis (Figure 2).

**Figure 3:**
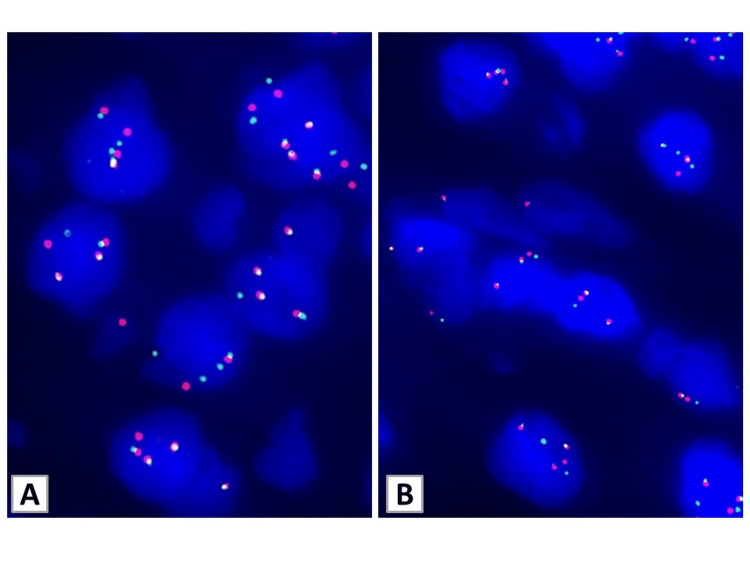
FISH results for ALK rearrangement In the biopsy specimens of lung (A) and bilateral breasts (B), FISH analysis using break-apart ALK probes detected ALK rearrangement showing isolated red and green signals along with fused red/green (normal) ones. Additionally, ALK copy number gain was noted in both specimens. ALK, anaplastic lymphoma kinase; FISH, fluorescence in situ hybridization

Meanwhile, a needle biopsy of bilateral breast tumors was performed, and the histological findings on HE staining corresponded to adenocarcinoma derived from the lungs or breasts. The IHC results were positive for TTF-1 and E-cadherin but were negative for estrogen receptor (ER), progesterone receptor (PgR), and human epidermal growth factor receptor 2 (HER2). Furthermore, ALK immunostaining was positive, and ALK rearrangement were detected with FISH analysis of lung and breast specimens (Figures 2, 3).

**Figure 4:**
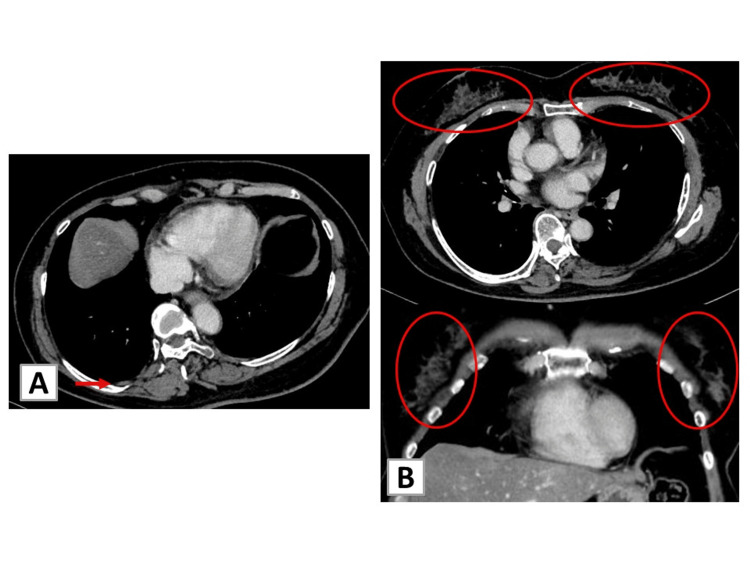
Chest CT findings at six months after the ALK–TKI treatment (A) CT showing tumor size reduction in the lower right lobe (arrow) and resolution of the pleural effusion. (B) CT in axial (upper) and coronal (bottom) views showing resolution of shadows in bilateral breasts (red circles). ALK, anaplastic lymphoma kinase; TKI, tyrosine kinase inhibitor

Based on those results and comparison with lung specimens, bilateral breast tumors were diagnosed as metastases from lung adenocarcinoma. Oral treatment with ALK-tyrosine kinase inhibitor (TKI), alectinib (600 mg once a day), was initiated, leading to a substantial reduction of tumor sizes to 1.2 cm. In the breasts, the metastases nearly disappeared and shrank to a maximum size of 4 mm (Figure 4).

Additionally, lymphadenopathy, pleural effusions, and pericardial effusions decreased. The CA 19-9 level decreased to 3.7 U/mL. This therapy is still ongoing, with no evidence of recurrence.

## Discussion

Lung cancer commonly metastasizes to lymph nodes, brain, liver, bones, and lungs. Breast metastasis from lung cancer is uncommon. In previous reports of breast metastasis due to lung cancer, differentiation from primary breast cancer or metastasis from other organs was challenging [6-8]. Among malignant breast tumors, the occurrence rate of metastatic breast tumors is low. Similarly, breast metastases from extramammary malignancies are infrequent, and the primary metastasis site in breast cancer is considered malignant melanoma (29.8%), lung cancer (16.4%), gynecological carcinoma (12.7%), or intestinal tumors (9.9%) [9]. Most previous reports regarding breast metastasis of lung cancer are about solitary metastatic tumors occurring unilaterally [10]; thus, the case of scattered bilateral breast metastases is rare.

When morphological differentiation and the confirmation of pathological diagnosis are challenging, IHC is valuable for the differential diagnosis of tumors. In lung adenocarcinoma, TTF-1 is typically found in a differential diagnosis. TTF-1 expression is most frequently seen in thyroid and lung carcinomas. In breast cancer, the ER, gross cystic disease fluid protein-15, mammaglobin, and GATA-3 are used in differential diagnosis [11]. In this case, positive immunostaining for TTF-1 in lung biopsy specimens corresponded to lung adenocarcinoma. On the contrary, the morphological differentiation of bilateral breast tumors did not also contradict invasive lobular carcinoma. The IHC for ER, PgR, and HER2 were negative in the immunostaining of bilateral breast specimens. However, based on the IHC positive results for TTF-1 and E-cadherin, bilateral breast tumors were resembled lung biopsy specimens.

ALK fusion genes were initially identified as anaplastic large-cell non-Hodgkin’s lymphoma [12]. Although anaplastic large-cell lymphoma and NSCLC are the most well-known ALK-associated tumors, they have been reported in various other types tumors in recent years, including breast tumors, inflammatory myofibroblastic tumors, neuroblastic tumors, histiocytosis, colorectal tumors, pancreatic tumors, and neuroendocrine tumors. Subsequently, many ALK fusion genes were identified in various tumors. [13]. The EML4-ALK rearrangements were initially described in 6.5% of Japanese patients with NSCLC [14]. For patients with NSCLC, the ODxTT system has been approved for the simultaneous detection of multiple gene alternations [15,16]. Our patient was diagnosed with lung adenocarcinoma based on IHC, and EML4-ALK was detected using the ODxTT in a lung biopsy specimen. Because examining with ODxTT in breast biopsy specimens is not approved for breast cancer in Japan, IHC and FISH were performed separately for ALK rearrangements because ALK expression was proved by IHC in the biopsy specimens of lung and breasts. The sensitivity of ALK protein detection was 85.7% and the specificity was 100% in IHC for ALK. Moreover, the IHC and FISH results generally showed a high correlation rate of >95%. This was followed by FISH analysis, which was employed with dual-probe hybridization [17]. FISH analysis was employed with dual-probe hybridization. ALK break-apart FISH probes were prepared from bacterial artificial chromosome clones RP11-984I21 and RP11-684O3 labeled with ENZO Orange-deoxyuridine triphosphate (dUTP) and ENZO Green-dUTP (Abbott Molecular Inc., Des Plaines, IL, USA), respectively. In the biopsy specimens of lung and bilateral breasts, FISH analysis using break-apart ALK probes detected ALK rearrangement, showing isolated red and green signals along with fused red/green (normal) ones. Additionally, ALK copy number gain was noted in both specimens. The presence of ALK rearrangement and copy number gain were deemed to be the mechanism of ALK inhibitor resistance [18].

ALK-positive lung cancers exhibit ALK dependency and are typically sensitive to ALK inhibition using TKIs such as crizotinib, ceritinib, alectinib, brigatinib, and lorlatinib [19]. Alectinib, given its relatively favorable response rate and low side effects, is used as one of the first-line drugs for patients with ALK-positive NSCLC [20]. In our case, despite ALK copy number gain, treatment with alectinib resulted in a significant reduction in pulmonary and bilateral breast tumors, with few side effects. Additionally, a marked decrease in CA19-9 levels was observed. Although CA19-9 is generally known as a marker for gastrointestinal cancers, it is also elevated in lung and breast cancer and benign disease.

These results indicate that the patient was diagnosed with ALK rearrangement-positive pulmonary adenocarcinoma with bilateral scattered breast metastases. Although hematogenous and lymphatic metastasis are possible, the mechanism of lung cancer metastasizing to breast is unknown. A previous report mentioned both mechanisms, stating that determining the diversity of cases is difficult [10]. Herein, hematogenous and lymphatic metastases were considered from other transition forms.

Limitations of the diagnosis of breast metastases of lung cancer are the similarity of the CT findings of multiple metastases of breast cancer and positivity rate of TTF-1. In scattered breast metastases, distinguishing between lung cancer and breast cancer on CT findings is challenging. Moreover, TTF-1 expression is most frequently seen in thyroid and lung carcinomas. The positivity rates of IHC for TTF-1 were 93% and 83%, respectively. However, 2.4% of breast cancer tests are positive. If histological similarities are present, close examination including other markers is required.

## Conclusions

Although bilateral breast metastases are rare, nodules in breasts are needed to be differentiated from mammary metastasis or malignant disease of other organs. This is a rare case that presented with bilateral scattered breast metastases from ALK rearrangement-positive pulmonary adenocarcinoma. If scattered breast masses with lung nodule are encountered, the possibility of breast metastasis from lung cancer should be considered.
